# A Study of the Polycondensation of (Tetrahydroxy)(Tetraaryl)Cyclotetrasiloxanes under Equilibrium and Non-Equilibrium Conditions in the Presence and Absence of Montmorillonite

**DOI:** 10.3390/polym10040422

**Published:** 2018-04-09

**Authors:** Nataliya N. Makarova, Yury I. Lyakhovetsky, Irina M. Petrova, Fedor M. Dolgushin, Nikolai S. Ikonnikov, Alexander S. Peregudov, Tatyana V. Strelkova, Zinaida S. Klemenkova

**Affiliations:** A. N. Nesmeyanov Institute of Organoelement Compounds of the Russian Academy of Sciences, 28 Vavilov St., 119991 GSP-1 Moscow, Russia; yulyakh@ineos.ac.ru (Y.I.L.); impetr2013@yandex.ru (I.M.P.); fedya@ineos.ac.ru (F.M.D.); ikonns@ineos.ac.ru (N.S.I.); asp@ineos.ac.ru (A.S.P.); o.strelkova@gmail.com (T.V.S.); zklem@ineos.ac.ru (Z.S.K.)

**Keywords:** polycondensation, equilibrium and non-equilibrium conditions, cyclolinear polyorganosilsesquioxanes, montmorillonite, cage-like compounds

## Abstract

Oligo- and polycyclosiloxanes were obtained by the polycondensation of (tetrahydroxy)(tetraaryl)cyclotetrasiloxanes in equilibrium and non-equilibrium conditions in the presence and absence of montmorillonite (MMT). Their composition and the structures of their components were investigated by infrared (IR) spectroscopy, ^29^Si nuclear magnetic resonance (NMR) spectroscopy, atmospheric pressure chemical ionization (APCI) mass spectrometry, powder X-ray diffraction (XRD), and gel-penetrating chromatography (GPC). Also, a comparison of polymers formed in the presence of MMT and via anionic polymerization was performed showing differences in their structures.

## 1. Introduction

The first hybrid cyclolinear (CL) ladder polyphenylsilsesquioxanes (LPPSSO) with unique physical and chemical characteristics were synthesized more than 50 years ago [1,2,3,4]. In one of the articles, the authors assumed that *cis*-*anti*-*cis*-tetraphenylcyclolsiloxane formed CL LPPSSO with the *cis*-syndiotactic “double-chain” structure, while the *cis*-*syn*-*cis* isomer under equilibrium polymerization gave cage-like structures consisting of 8–12 PhSiO_1.5_ groups [5]. This indicates that steric parameters of a monomer can substantially affect the results of polymerization or polycondensation processes of organocyclosiloxanes. Due to the hydrodynamic and electro-optical characteristics of LPPSSO solutions, the differences in the α-values in the Mark–Kuhn–Houwink equation and the Kuhn segment were revealed depending on the ladder polymer structure, production method, organic substituents at the silicon atoms, and the structure of the prepolymer [6,7,8,9]. Despite the fact that 50 years have passed, interest in the steric characteristics of the polymers, these hybrid CL LPPSSO and other CL ladder polyorganosilsesquioxanes (LPOSSO), persists. This is because LPOSSO are known to be highly thermostable, to possess good mechanical parameters, and to be capable of forming thin films [1,7,10]. These properties apparently depend on the structure and the steric characteristics of the polymers. 

Stereoregular LPOSSO were synthesized using organotrifunctional silanes (organotrichloro- or organotrialkoxysilanes) [10,11,12]. *Cis*-isotactic CL LPPSO were prepared by polycondensation of 1,1,3,3-dihydroxy-1,3-diphenyl-1,3-disiloxane either on a glass surface or in acetonitrile solution [13]. The authors also obtained *cis*-isotactic LPOSSO via polycondensation of 1,1,3,3-tetrahydroxy-1,3-diorgano-1,3-disiloxane in the presence of Me_4_NOH as a catalyst, a π-stacking and H-bonding superstructure of the monomer promoting the reaction [14]. Polycondensation of *cis*-*trans*-*cis*-tetrahydroxytetramethylcyclosiloxane in the presence of K_2_CO_3_ furnished *cis*-syndiotactic polymethylsesquioxanes with the MeSIOH groups located in the chain between the polymethylsilsesquioxane blocks [15].

The processes of polycondensation of *cis*-*anti*-*cis*-(tetrahydroxy)(tetramethyl)cyclotetrasiloxane and all-*cis*-tetrahydroxy)(tetravinyl)cyclotetrasiloxane were also investigated [15,16]. Attention was paid to the study of the influence of the conditions of polycondensation and stepwise coupling polymerization of these organocyclotetrasiloxanes upon the composition and structure of the CL polymethylsilsesquioxanes and ladder polyvinylsilsesquioxanes (LPViSSO) formed [17,18,19]. The reactions of stepwise condensation of 2,4,6,8-(tetrahydroxy)(tetra-*i*-propyl)cyclotetrasiloxanes and 1,3-dichloro-1,3-di-*i*-propyl-1,3-diphenyldisiloxane were employed to obtain stereoregular penta-, hepta-, and nona-cycle ladder siloxanes [20,21,22]. 

**Figure 1:**
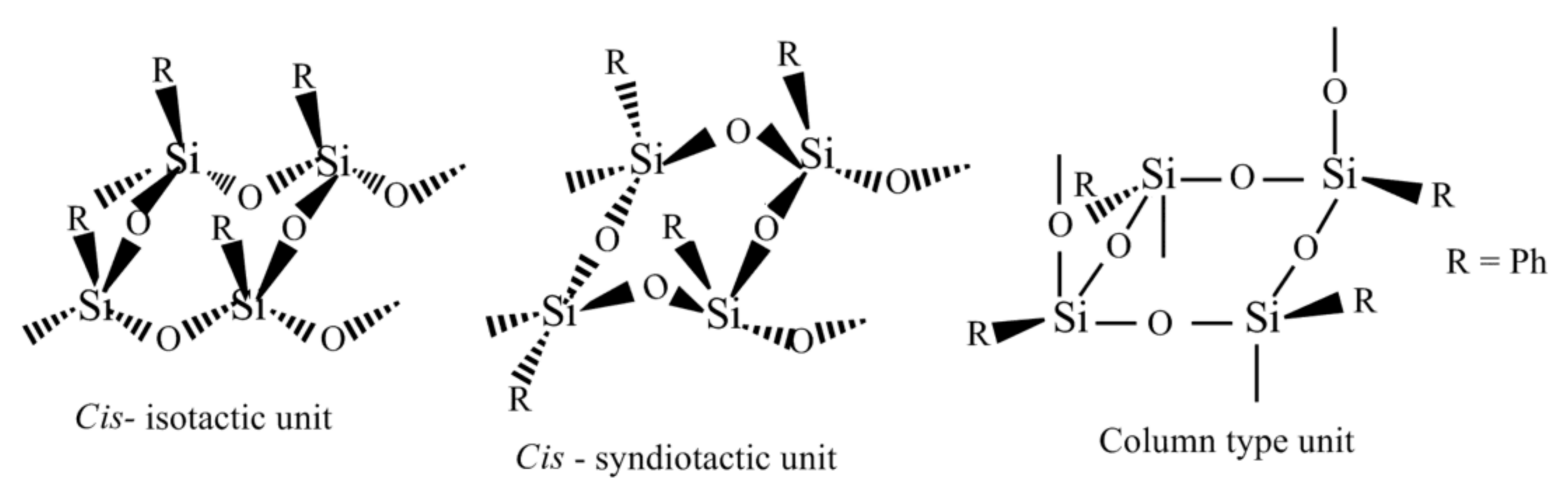
Structures of units in cyclolinear ladder polyphenylsilsesquioxanes (CL LPPSSO).

We recently reported polycondensation of all four isomers: all-*cis*, *cis*-*trans*-*cis*-, *cis*-*cis*-*trans*-, and all-*trans*-(tetrahydroxy)(tetraphenyl)cyclotetrasiloxanes [PhSi(OH)O]_4_ by another method, namely, in the presence of layered-architecture compounds. The formation of LPPSSO with different unit structures and column-type cyclolinear polyphenylsilsesquioxanes was observed [23,24]. For example, by means of copolycondensation of the all-*cis*- and *cis*-*trans*-*cis*-stereoisomers of [PhSi(OH)O]_4_ in solution in the presence of montmorillonite (MMT) in various ratios to the isomers, LPPSSO copolymers were obtained [25] Only two isomers of the four possible isomers, all-*cis*- and *cis*-*trans*-*cis*-[PhSi(OH)O]_4_, out formed the *cis*-isotactic and *cis*-syndiotactic CL PPSSO structures, respectively. However, in the case of the all-*cis* isomer when the reactions were conducted both with and without MMT cage-like oligomers also formed. With that, the *cis*-*trans*-*cis* isomer formed CL LPPSO with a higher M_w_ than the all-*cis* one. The all-trans isomer furnished column-type CL PSSO (Figure 1).

Thus, the configuration of the starting monomer affected the structure of the units of the polymer formed. The introduction of a substituent into the phenyl ring can impact on the configuration in which the starting (tetrahydroxy)(tetraaryl)cyclotetrasiloxane is formed when synthesized and thus can influence the structures of oligomers and polymers obtained via the polycondensation reaction. It is especially so when the reaction implies intercalation of the monomer into a layered-architecture compound like MMT. 

With this in mind, we have synthesized two compounds, stereoisomers of 2,4,6,8-tetrahydroxy-2,4,6,8-tetra-*m*-tolyl- and 2,4,6,8-tetrahydroxy-2,4,6,8-tetra-*m*-chlorophenyl-cyclotetrasiloxanes (**1** and **2a**, respectively) containing substituents at phenyls of different types, donating (Me) and mixed (Cl), and report here on their polycondensation reaction performed under different conditions and in the presence and absence of MMT. A mixture of two isomers of the latter compound (**2b**) was also prepared and polycondensation of it was investigated.

## 2. Experimental Section

### 2.1. Materials

Stereoisomer **1** was synthesized by hydrolysis of trichloro-*m*-tolylsilane with additional recrystallization following a published procedure [26]; m.p. 160 °C, *R*_f_ = 0.05 (TLC, toluene: diethyl ether 1:1), ^29^Si nuclear magnetic resonance (NMR) (acetone-D_6_) (δ ppm): −70.16 (s), and atmospheric pressure chemical ionization (APCI) mass spectrum: 626 (nominal mass) [M + H_2_O]^+•^ Compound **2a** (pure isomer) was prepared by hydrolysis of trichloro-*m*-chlorophenylsilane by method [27]; m.p. > 350 °C, *R*_f_ = 0.50 (TLC, toluene: diethyl ether 1:1), and ^29^Si NMR (acetone–D_6_) (δ ppm): −71.81 (s). Compound **2b** (a mixture of two isomers with a ratio of 45:55) was prepared by hydrolysis of trichloro-*m*-chlorophenylsilane by a modified method [26] at a concentration of HCl 0.035 mol·L^−1^; ^29^Si NMR (acetone–D_6_) (δ ppm): −71.61 (s) and −71.82 (s), ^1^H NMR (acetone–D_6_) (δ ppm): 6.73 (s), 6.75 (s) (OH), and *R*_f_ = 0.05 and 0.50 (TLC, toluene: diethyl ether 1:1). The former *R*_f_ was the same as for **1**, while the latter for **2a**. Both *R*_f_ values coincided with those obtained for all-*cis*- and *cis*-*trans*-*cis*-tetrahydroxytetraphenylcyclotetrasiloxanes under the same TLC conditions, respectively [23]. This allowed for the suggestion that **1** had the all-*cis* configuration and **2a** was the *cis*-*trans*-*cis* isomer, while mixture **2b** comprised the all-*cis*- and *cis*-*trans*-*cis* isomers.

### 2.2. Measurements

^1^H and ^29^Si NMR spectra were recorded on a Bruker AV-600 spectrometer (Bruker Corporation, Karlsruhe, Germany) in aceton-D_6_ or CDCl_3_ solutions at 20 °C. IR spectra were obtained with a Specord M-82 Bruker spectrophotometer (Bruker Corporation, Ettlingen, Germany) using KBr pellets and in CCl_4_ solutions. 

The average molecular masses and the molecular mass distributions were determined by gel-penetrating chromatography (GPC) (Waters Corporation, Milford, MA, USA) using a Waters instrument comprising an M601 pump, an M-484 UV-Vis detector (λ = 260 nm), an M-410 refractometric detector for two U-Styragel linear columns, and the Millennium data processing system. THF (Sigma-Aldrich, Saint Louis, MO, USA) was used as a solvent at a flow rate of 1 mL·min^−1^ at 30 °C. 

Atmospheric pressure chemical ionization (APCI) mass spectra were measured on a Thermo Finnigan LCQ Advantage tandem dynamic mass spectrometer ( San Jose, CA, USA) equipped with an octapole ion trap mass analyzer, a Surveyor MS pump, and a nitrogen generator Schmidlin-Lab., model N2 Mistral-4 (Schmidlin-Lab, Neuheim, Switzerland). Nitrogen was served as sheath and auxiliary gases. The vaporizer temperature was 400 °C and the flow rate of acetonitrile was 350 μL·min^−1^. The temperature of the heated capillary was 150 °C; the corona discharge current was 5 μA. Samples dissolved in a chloroform:acetonitrile solution (2:5) were introduced into the ion source through a Reodyne injector with a 5 μL loop. The data collection and treatment were fulfilled using the program Xcalibur, version 1.3.

Powder X-ray diffraction (XRD) patterns of the compounds under study were collected using a Bruker D8 Advance Vario diffractometer (Bruker Corporation, Karlsruhe, Germany) with copper radiation, Kα_1_ monochromator (wavelength 1.54184 Å), and a LynxEye position sensitive detector in the 2θ range from 0.8° to 80° with an increment of 0.01° in 2θ. The patterns were analyzed using the TOPAS software (Bruker Corporation, Karlsruhe, Germany). 

### 2.3. Preparation of Products ***3.1***, ***3.2***, ***4***, and ***5***, and Polymers ***6**–**9***

#### 2.3.1. Preparation of Products **3.1**, **3.2**, **4**, and **5**

Isomer **1** (0.040 g, 0.66 × 10^−4^ mol) was dissolved in toluene (0.16 mL). The solution was boiled in a molybdenum glass flask at 110 °C for 2 h. The polycondensation reaction was monitored by IR, ^1^H NMR spectroscopy of OH groups, and positive ion mode APCI mass spectrometry (PI APCI MS). Water was removed from the reaction mixture into a Dean-Stark trap. The solvent was removed in vacuum until a constant weight of the residue. Product **3.1** (0.035 g), was obtained in *ca* 93% yield.

Product **3.2** (0.035 g) was prepared in a similar manner by condensation of **1** in a quartz flask; the yield was *ca* 93%. The IR, ^29^Si NMR spectra, and the PI APCI mass spectra of products **3.1** and **3.2** are depicted either in the Results and Discussion section or in the Supplementary Materials (SM). 

Isomer **1** (0.040 g, 0.66 × 10^−4^ mol) was dissolved in ditolylmethane (0.19 mL). The solution was boiled in a molybdenum glass flask in vacuum 1 Torr at 95 °C for 2 h. The polycondensation reaction was monitored by infrared (IR), ^1^H NMR spectroscopy, and PI APCI MS. The solvent was removed in vacuum until a constant weight of the residue. Product **4** (0.035 g), was obtained in *ca* 93% yield. The corresponding IR, ^29^Si NMR spectra, and PI APCI mass spectra are given below or in the SM.

Isomer **1** (0.040 g, 0.66 × 10^−4^ mol) was dissolved in toluene (0.13 mL), and MMT (0.012 g) was added. The solution was boiled in a molybdenum glass flask at 110 °C for 2 h. The polycondensation reaction was monitored by IR, ^1^H NMR spectroscopy until almost complete consumption of the OH groups, and by PI APCI MS. Water was removed from the reaction mixture into a Dean-Stark trap. MMT was filtered off, and the solvent was removed in vacuum until a constant weight of the residue. Product **5** (0.035 g), was obtained in *ca* 93% yield. The IR, ^29^Si NMR spectra, and PI APCI mass spectra of **5** are given below and in the SM. 

#### 2.3.2. Synthesis of Polymers **6**–**8**

Polymers **6** (0.033 g) and **7** (0.017 g) were synthesized in molybdenum glass flasks under equilibrium and non-equilibrium reaction conditions (in toluene at 110 °C and in vacuum of 1 Tor in ditolylmethane at 95 °C, respectively) by protocols similar to that described below to obtain polymer **8** but without adding MMT. The yields were 95% and 87%, respectively. 

Compound **2a** (0.084 g, 1.22 × 10^−4^ mol) was dissolved in anisole (0.28 mL), and MMT (0.024 g) was added. The solution was boiled in a molybdenum flask at 110 °C for 2 h. The polycondensation reaction was monitored by IR and ^1^H NMR spectroscopy until complete consumption of the OH groups. MMT was filtered off, and the solvent was removed in vacuum until a constant weight of the residue. Polymer **8** (0.067 g), was obtained in 84% yield. *M*_w_ was 2000–75,000 (GPC), *d*_1_ = 14.40 Å. The IR and ^29^Si NMR spectra of **8** are depicted below.

Compound **2b** (0.041 g, 0.60 × 10^−4^ mol) was dissolved in anisole (0.14 mL), and MMT (0.012 g) was added. The further procedure was similar to the one described above for **2a**. Polymer **9** (0.029 g) was obtained in 76% yield. *M*_w_ was 2000–49,000 (GPC), *d*_1_ = 14.40 Å. For the IR and ^29^Si NMR spectra of **9** see below. 

## 3. Results and Discussion

Polycondensations of compounds **1**, **2a**, and **2b** were carried out in equilibrium (under atmospheric pressure) and non-equilibrium (in vacuum) conditions in toluene, anisole or ditolylmethane in quartz or molybdenum glass flasks in the absence and in the presence of MMT (see Note 1 in the SM).

The reactions were monitored by GPC, PI APCI MS, IR, and ^1^H NMR spectroscopy methods. 

**Figure 2:**
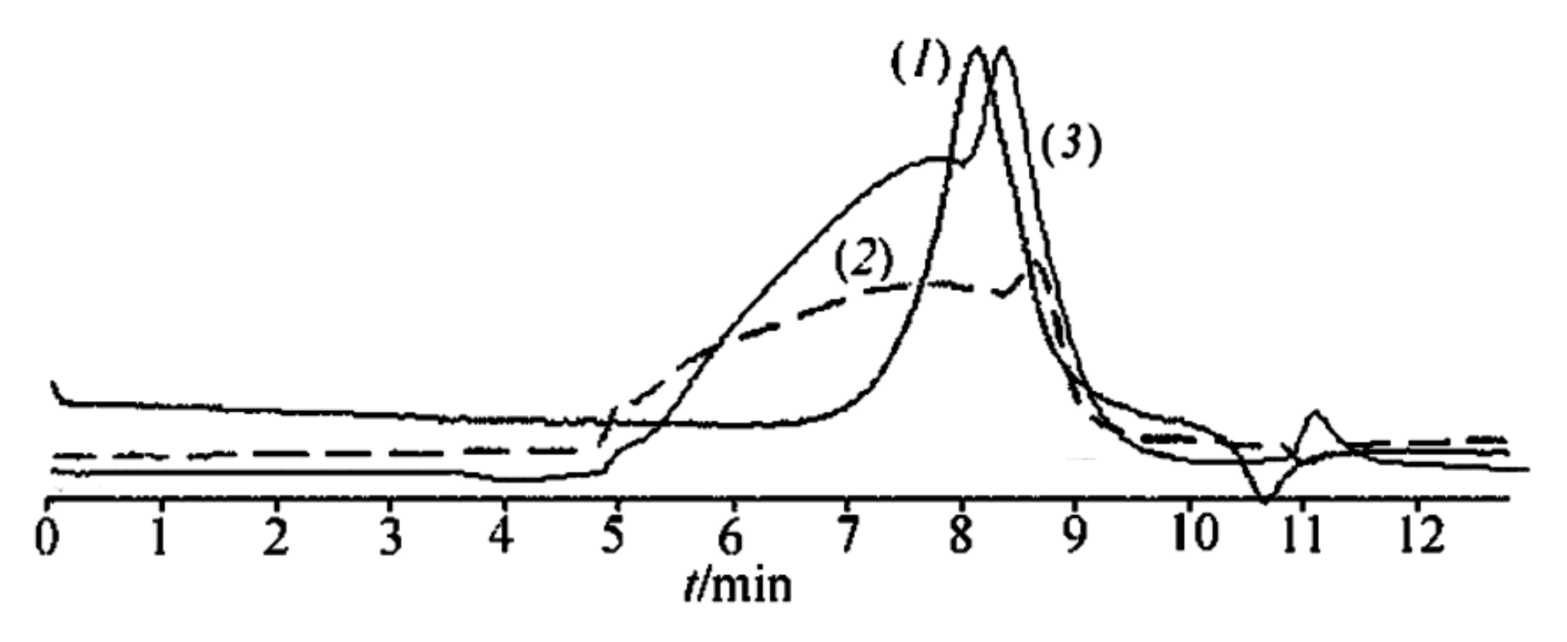
Gel-penetrating chromatography (GPC) tracks of LPOSSOs: (*1*) of product **5**, (*2*) of polymer **8**, and (*3*) of polymer **9**.

Products **3.1**, **3.2**, and **4** obtained after the same time period of 2 h from polycondensation of **1** in toluene solution at atmospheric pressure or in vacuum in ditolylmethane, respectively, had similar values of molecular weight *M*_w_ = 1900–2800 and *M*_w_/*M*_n_ = 1.2–1.9 according to GPC (Figure 2). For product **5** obtained in a molybdenum glass flask at atmospheric pressure in the presence of MMT, the same above values were observed (see Figure 2, curve (*1*) for the experimental GPC track). These data show that the reactions of polycondensation of **1** in equilibrium and non-equilibrium conditions afforded oligomeric products. 

These results are in good agreement with those previously reported for polycondensation of the all-*cis*-isomer of [PhSi(OH)O]_4_ under analogous conditions in the absence of MMT [23].

Moreover, curve (*2*) in Figure 2 totally coincided in shape and molecular weight interval with that for CL LPPSSO obtained from polycondensation of *cis*-*trans*-*cis*-[Ph(OH)SiO]_4_ in anisole in the presence of MMT [23]. This provided an additional argument in favor of the aforesaid suggestion that **2a** was the *cis*-*trans*-*cis*-isomer. 

Polycondensation of compound **2a** in the absence of MMT under both equilibrium and non-equilibrium conditions led to polymers **6** and **7**, respectively, with *M*_w_ = 5200; *M*_w_/*M*_n_ = 1.7.

**Figure 3:**
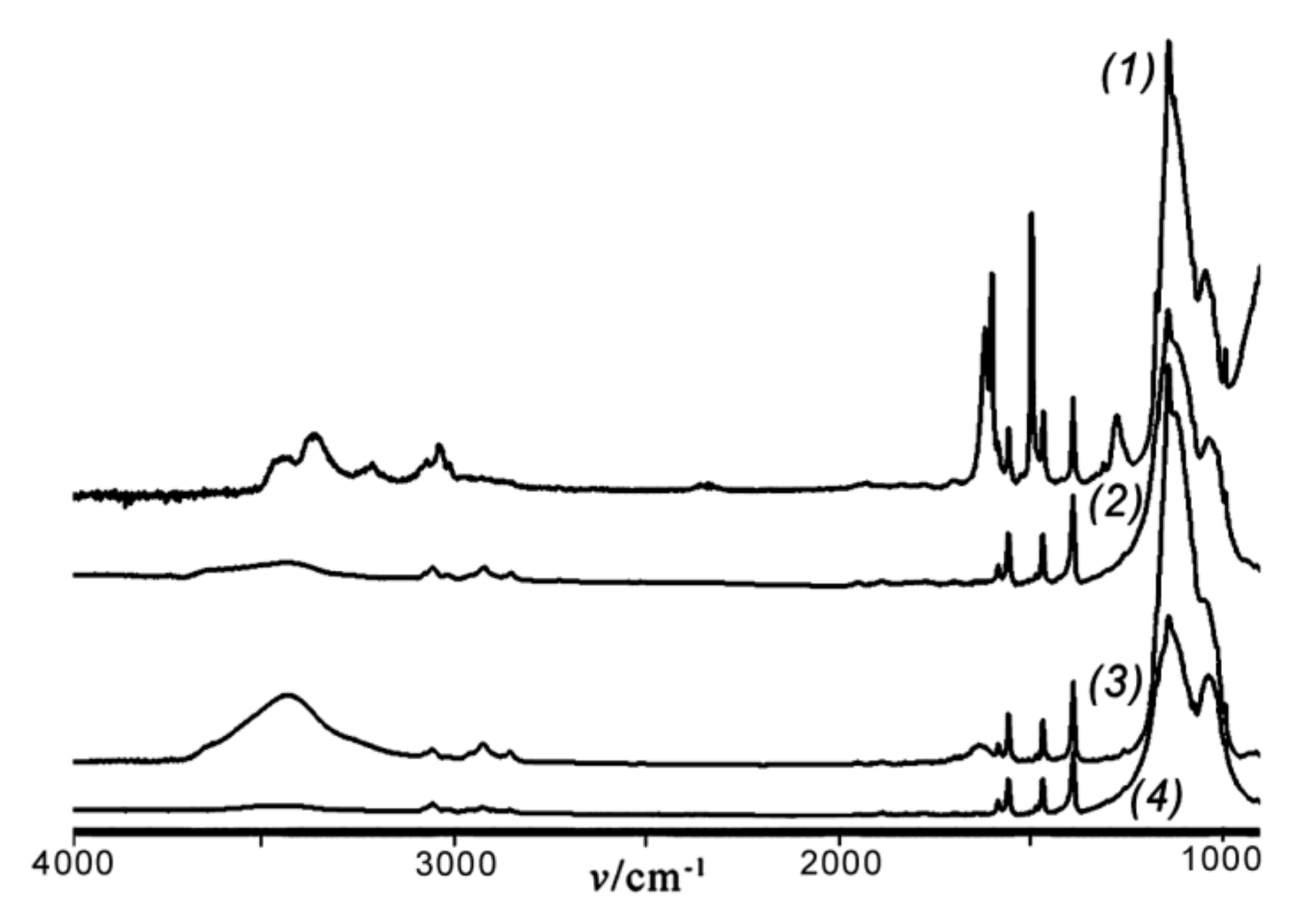
Infrared (IR) spectra of polymer **7** (*1*), polymer **8** (*2*), polymer **9** (*3*), and polymer **10** (*4*).

In contrast to these products, polymer **8** obtained by polycondensation of compound **2a** in anisole in the presence of 29% MMT had *M*_w_ in the range 2000–75,000 after the same time period of 2 h (Figure 2, curve (*2*)). Polycondensation of compounds **2b** under identical conditions over the same time period led to the formation of polymer **9** (see Figure 2, curve (*3*) for the experimental GPC track). A comparison of curves (*2*) and (*3*) in Figure 2 showed that the use of the **2b** mixture for thermal polycondensation led to an increase in the weight fraction of the oligomeric product and to a slight decrease in the molecular weight of the polymer. Moreover, the presence in **2b** of another isomer with *R*_f_ = 0.05 besides that with *R*_f_ = 0.50 (see the Experimental section) gave rise to the formation of a cyclolinear ladder polymer with different configuration of the units. This is seen from the IR spectra of polymers **8** and **9** ((*2*) and (*3*) in Figure 3, respectively), the different configuration of the units in **9** as compared to that of **8** being indicated by different forms of (*3*) and (*2*) in the 1070–1000 cm^−1^ region. As for polymer **8**, the IR spectrum of polymer **7** (curve (*1*) in Figure 3) obtained by polycondensation of **2a** in vacuum and in the absence of MMT displays two bands at 1050~1055 and 1150~1155 cm^−1^ which indicate the formation of CL PClPSSO also in this case.

No reaction was observed, when **2a** was heated with MMT at 110 °C without solvent. Low-molecular-weight polymers were obtained from the reaction in toluene at 110 °C in the presence of 5–20% *w*/*w* % of MMT in respect to **2a**. Only when the addition of MMT was increased to 29%, the polycondensation (in anisole at 110 °C) afforded polymer 8 with *M*_w_ up to 75,000 (see above). Thus, the reaction in the presence of these amounts of MMT allowed obtaining a polymer the molecular weight of which was higher than the molecular weight of polymer **6** obtained in the absence of MMT. 

The role of MMT lay probably in the fact that intercalation of hydroxycyclotetrasiloxane **2a** from the solution into galleries between MMT layers resulted in the disruption of intermolecular hydrogen bonds in **2a**. This led to self-organization of the **2a** molecules on the MMT surface that facilitated polycondensation, thus providing the increased length of the polymer chain in **8** as compared with that in **6**.

**Figure 4:**
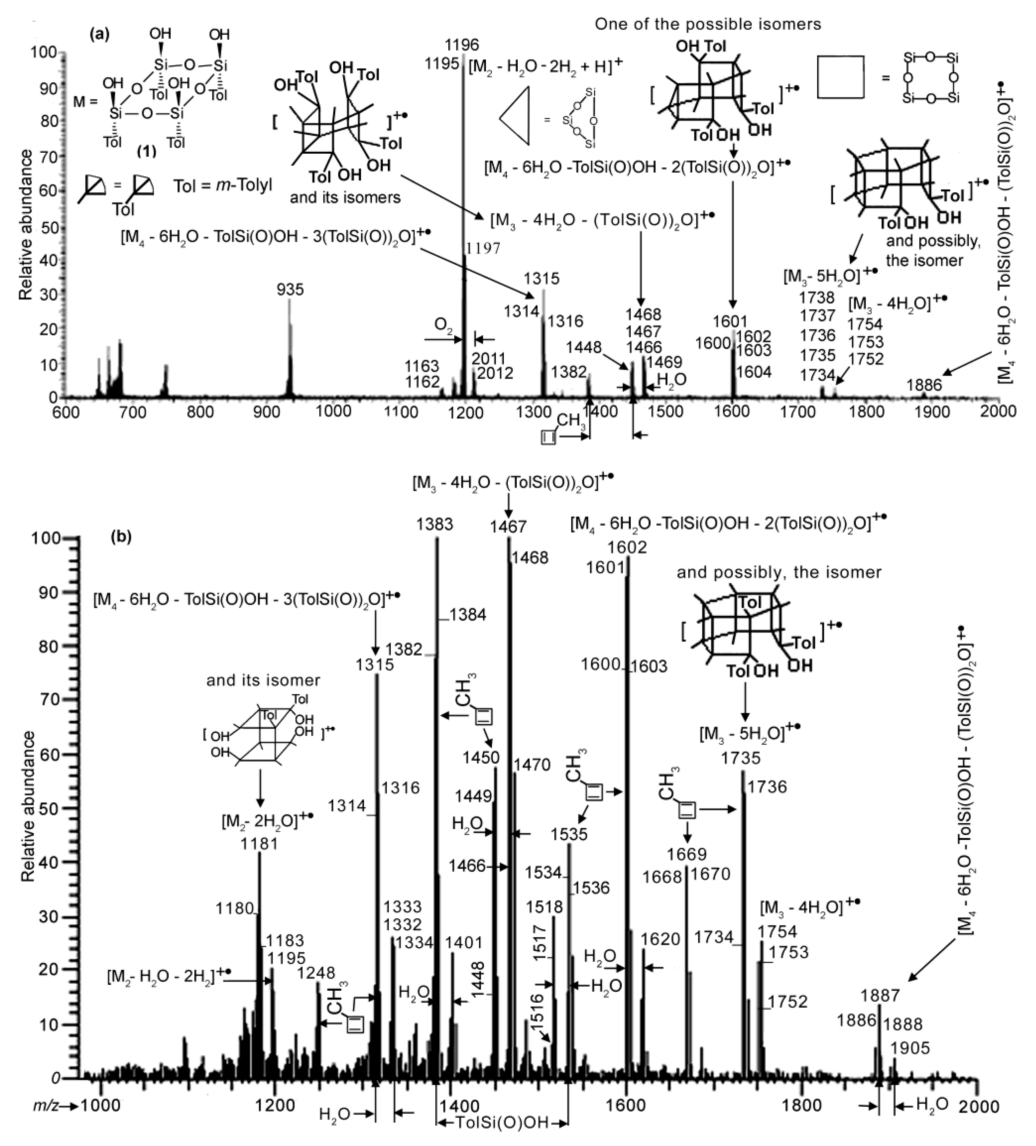
Positive ion mode atmospheric pressure chemical ionization (PI APCI) mass spectra of the products obtained after heating of a toluene solution of **1** at atmospheric pressure at 110 °C: (**a**) in a quartz flask for 130 min (product **3.2**) and (**b**) in a molybdenum glass flask for 120 min (product **3.1**); the schemes at the peaks mostly give the routes of the formation of the corresponding compounds rather than fragmentation paths in the mass spectrometer. However, some contributions from the fragmentation processes are possibly present in ions, these fragmentations probably occurring as ‘in-source collision-induced dissociation’ [28,29] (also, see Note 2 in the Supplementary Materials (SM)).

**Figure 5:**
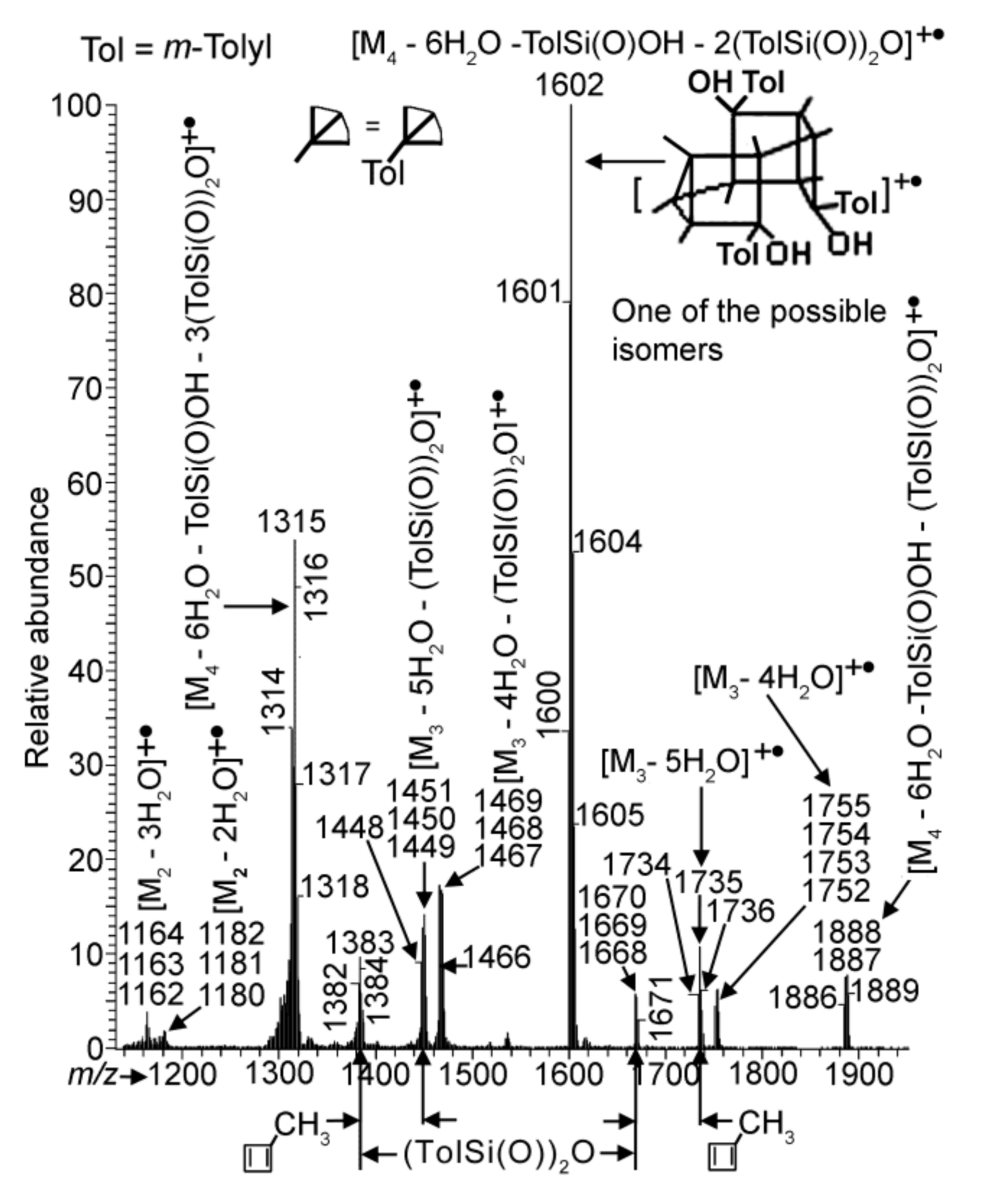
PI APCI mass spectrum of the products obtained after heating of a toluene solution of **1** at atmospheric pressure at 110 °C in a molybdenum glass flask in the presence of MMT for 120 min (product **5**).

As mentioned above, polycondensation of compound 1 under different conditions (at atmospheric pressure or in vacuum with or without MMT) was monitored by APCI MS. Figure 4 and Figure 5 demonstrate the APCI mass spectra of products **3.2**, **3.1** and **5** obtained after heating of compound **1** in quartz and molybdenum glass flasks, and in a molybdenum glass flask in the presence of MMT, respectively, all at atmospheric pressure and 110 °C for comparable periods of time. All three spectra show species formed via condensation between two and four molecules of compound **1**. With that, the spectra demonstrate the loss of (TolSi(O))_2_O (Tol = *m*-tolyl) and sometimes of TolSi(O)OH, the former being preliminarily reported for the phenyl analogue [17,24]. This loss most likely occurred in the course of the reactions rather than in the mass spectrometer as fragmentations. Ion peaks corresponding to compounds with the nominal masses 1180 Da for **3.1** and 1162 and 1180 Da for **5** appeared owing to the condensation of two molecules of **1** (single condensation) could also be considered as belonging to the species formed via the decomposition of double condensation products (products of condensation of three molecules of **1**) according the schemes (M_3_ − 5H_2_O) − 2(TolSi(O))_2_O and (M_3_ − 4H_2_O) − 2(TolSi(O))_2_O. However, the relative abundances of the peak group with the *m*/*z* 1180 ion decreased strongly in time (*cf*. those in Figure S2a,b in the SM, and Figure 4b). This suggests that the compounds discussed were formed via the condensation of two molecules of **1**.

Nevertheless, spectra (a) and (b) of Figure 4 and that of Figure 5 differ from each other. In the first spectrum, the most abundant group of peaks is at *m*/*z* 1195–1197. It belongs to a species formed due to the single condensation process, while ion peaks of the species obtained via double condensation and condensation of four molecules of **1** (triple condensation) are significantly less intensive. Moreover, spectra S1a–c in the SM and spectrum 4a give the formation dynamics of product **3.2**. In the spectrum recorded after heating of **1** for 30 min, the most abundant peaks turned out to be those of species formed owing to the triple condensation process (at *m*/*z* 1315 and 1601). When **1** was heated for 60 min, a peak at *m*/*z* 1196 of a single condensation product became maximal and among the less-abundant peaks of the double and triple condensation species the peak of the former species at *m*/*z* 1383 was the greatest in intensity. This pattern was retained for the 90 min spectrum, while at last after 130 min, as mentioned above, the single condensation species became strongly dominant (see Figure 4a). A reasonable explanation for such a behavior is that, initially, the reaction was kinetically controlled. Then, after water accumulated in sufficient amounts in the reaction mixture due to the removal from the reacting species, the thermodynamic control came into play with the single condensation species as the major product.

Another situation was realized when the reaction was carried out in a molybdenum glass flask. It was previously shown that polymerization (and apparently, condensation) could be catalyzed by ions from glass [30,31]. Probably, the equilibrium was achieved here at higher rates of the direct reactions and as a result, double and triple condensation products proved to prevail (see Figure 4b and Figure S2a,b in the SM).

For the reaction in the molybdenum glass flask in the presence of MMT, the following should be highlighted. The process gave species of triple condensation as the main components of the overall product **5** (Figure 5). Contrary to spectrum (b) of Figure 4 (for the reaction without MMT), spectrum 5 exhibits peaks of only two relatively abundant ionic groups, one with the nominal mass ion 1314 Da and another with 1600 Da, peaks of all other ionic groups being significantly less intensive. The most abundant group in the spectrum is of 1600 Da ion, and a possible structure of the corresponding compound is furnished in Figure 5. It is worth noting that no essential changes were found when the reaction in the presence of MMT was conducted in anisole at 150 °C for 120 min (see Figure S3a in the SM). 

An interesting point of the spectra is worth discussion here. Monomer **1** was registered in the PI APCI spectrum as (M + H_2_O)^+•^ rather than M^+•^ (see the Experimental section), i.e., water was added immediately in the mass spectrometer. Whether or not this is valid for other compounds registered remains questionable. To throw some light on this, product **5** was treated with an excess of Me_3_SiCl and a mass spectrum recorded (Figure S3b in the SM). A shift by 72 Da to the higher masses was observed for the ionic groups with the nominal mass ions of 1314 and 1600 Da to give those of 1386 and 1672 Da. This indicated that the corresponding compounds contained a free OH group. At the same time, the groups with the nominal mass ions 1448 and 1734 proved to be unshifted. This speaks in favor of these compounds being closed-cage species bearing no OH groups, and water was added during the mass spectral analysis. The corresponding ions could have either hydrate structures: [((M_3_ − 6H_2_O) − (TolSi(O))_2_O) + H_2_O]^+•^ and [(M_3_ − 6H_2_O) + H_2_O]^+•^, respectively, or hydrolysis of a Si–O bond occurred to furnish [M_3_ − 5H_2_O − (TolSi(O))_2_O)]^+•^ and [M_3_ − 5H_2_O]^+•^ with two adjacent OH groups. The same is probably valid for many other ions. For simplicity, the second variant is mostly employed in the labels in the mass spectra and the structures depicted therein. 

As could be anticipated, the reaction conducted in a vacuum (under non-equilibrium conditions) afforded a mixture of compounds formed due to single, double, and triple condensations of **1**, the two last-mentioned groups prevailing with many their components being of relatively high abundance in the APCI mass spectra (see Figure S4 in the SM). Another interesting point is that an ionic group containing the nominal mass ion of 1734 Da is of a pronounced abundance in all vacuum reaction spectra. This belongs to a species (M_3_ − 5H_2_O). In all other APCI mass spectra its peaks turned out to be of relatively less abundance. This is consistent with the fact that the decomposition of the compound occurred with the participation of water, whereas most of the evolving water was removed from the reaction in the vacuum process.

Peaks of the compound with the nominal mass of 1448 Da are also of interest. In Figure 4 and Figure 5 they are not among the most abundant. However, when the reaction was performed in a vacuum, this compound turned out to be dominant (see Figure S4 in the SM). As was revealed before, it could most probably have a closed-cage structure with a water molecule added in the course of the analysis. Moreover, the corresponding radical cation could again be either a closed-cage hydrate, or an open-cage species with two OH groups formed due to hydrolysis. Both variants are depicted in Figure S4c,d in the SM (also, see Note 3 in the SM).

Unfortunately, our attempts to collect the APCI mass spectra for the products of the polycondensation of compound **2** failed (see Note 4 in the SM).

**Figure 6:**
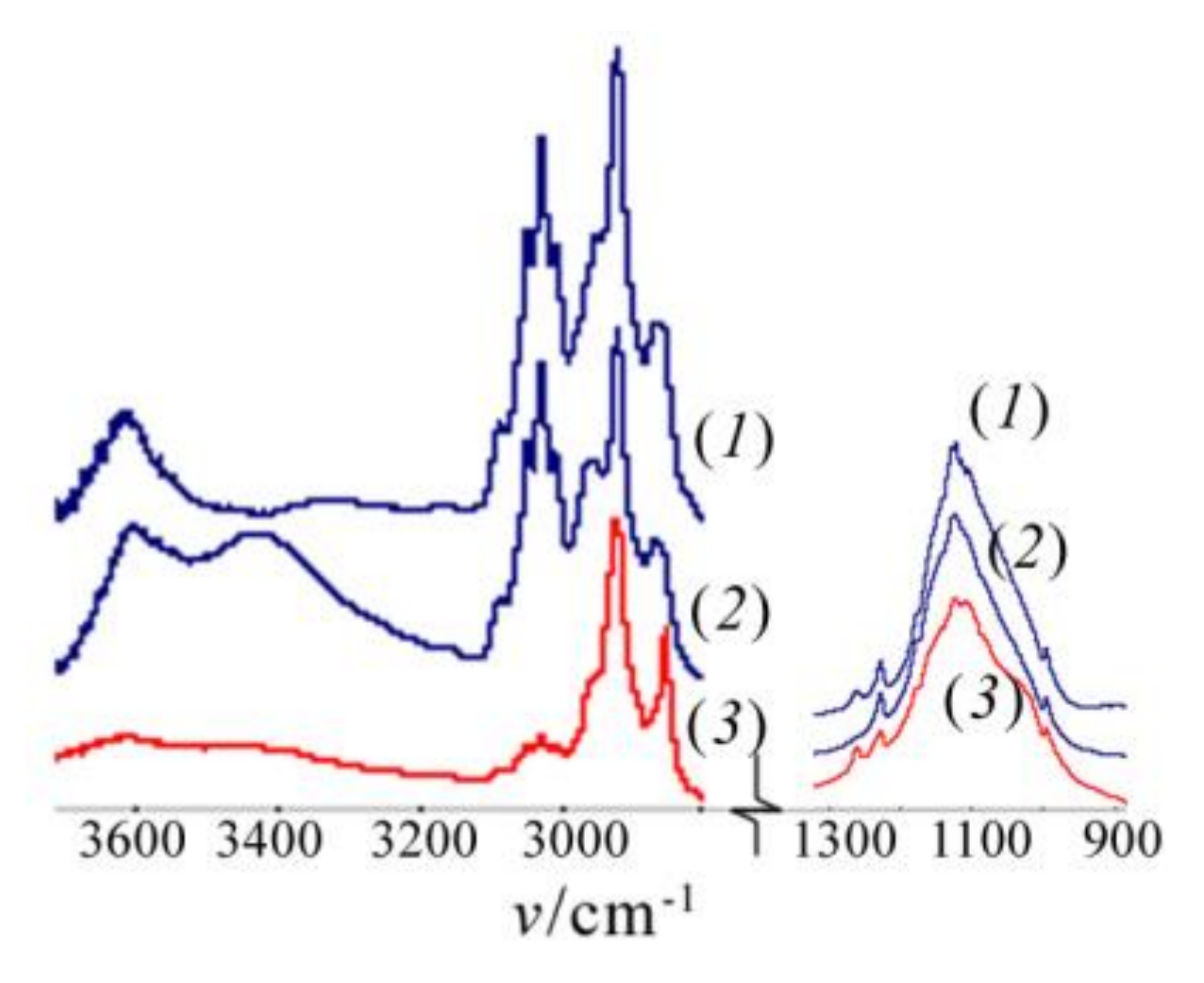
IR spectra of products **3.1**, **4**, and **5**: (*1*) **3.1**, (*2*) **4**, and (*3*) **5**.

The IR spectra of products **3.1**, **4**, and **5** (Figure 6) exhibited an absorption band at 1123 cm^−1^ with a width ∆*ν*_1/2_ of 200 cm^−1^ in the region of asymmetrical stretching vibrations *ν*_as_ of the Si–O–Si bond. The IR spectra of products **3.1** and **4** also exhibited noticeable OH bands at 3400–3600 cm^−1^, while that of **5** was less pronounced. This supported the above MS results of the presence in these products of open-cage compounds with OH groups. Moreover, this is in good agreement with the findings that cage-like organosilsesquioxanes were characterized by an absorption band at 1122–1128 cm^−1^ [5]. 

In contrast to the IR spectra of products **3.1**, **4** and **5**, those of polymers **7** and **8** obtained via polycondensation of compound **2a** exhibited two absorption bands *ν*_as_ of the Si–O–Si bonds at 1037–1040 cm^−1^ and 1140–1143 cm^−1^ with ∆*ν*_1/2_ of ~200 cm^−1^ (Figure 3). The spectra of **6**, **7**, and **9** showed OH absorption bands at 3300–3600 cm^−1^, whereas that of polymer **8** did not. 

**Figure 7:**
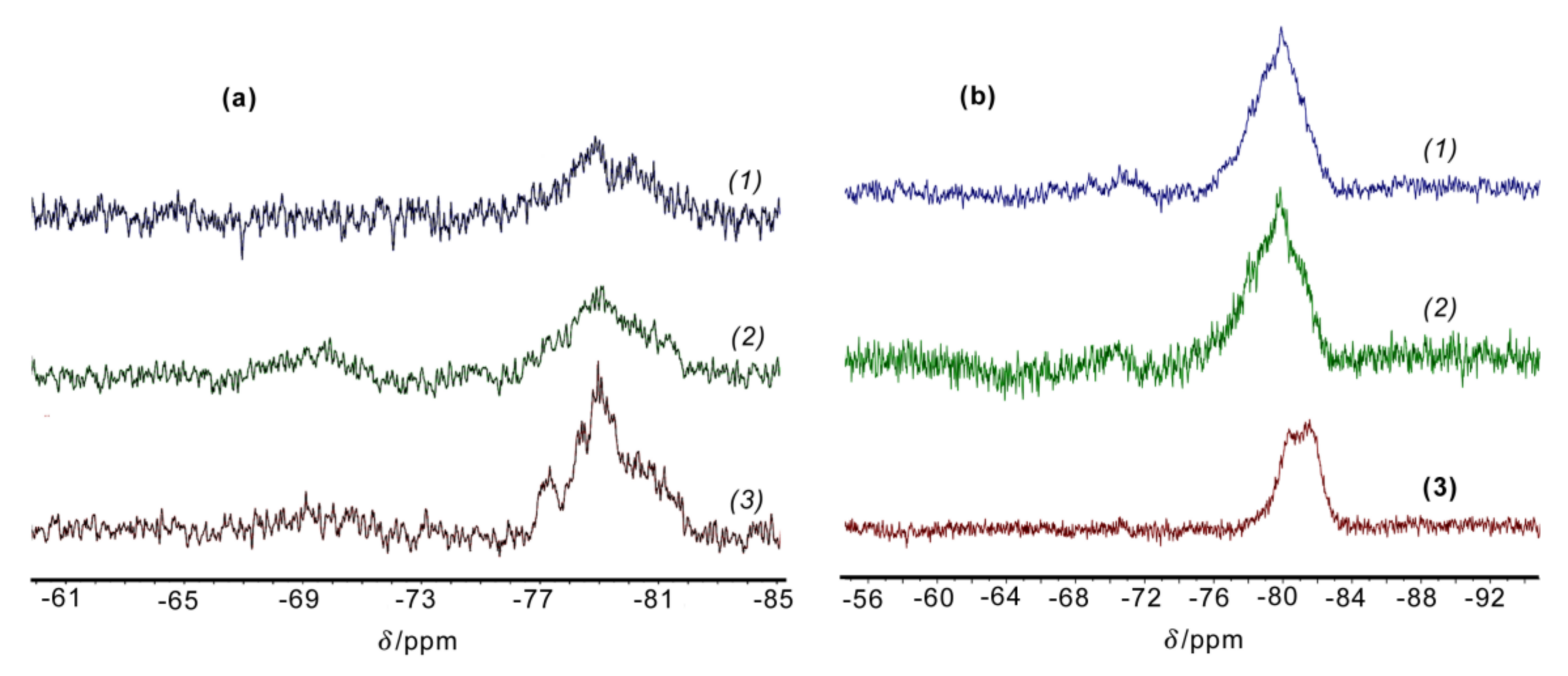
^29^Si nuclear magnetic resonance (NMR) spectra: (**a**) (*1*) of product **3.2**, (*2*) of **4**, and (*3*) of **5**; and (**b**) (*1*), (*2*), and (*3*) of polymers **8**, **9**, and **10**, respectively.

The ^29^Si NMR spectra of products **3.2**, **4**, and **5**, and polymers **8** and **9** were characterized by signals in two regions at δ −67.0~−71.0 (excluding **5**) and at δ −76.0~−82.0 ppm with different intensities and half-widths (Figure 7). The spectra of polymers **6** and **7** were similar to that of **8**. For products **3.2** and **4**, multiplets at δ −67.0~−71.0 ppm were weak in intensity, whereas those at δ −76.0~−82.0 ppm were more intensive and very broad. The ^29^Si NMR spectrum of product **5** totally coincided with the ^29^Si NMR spectrum of the products obtained by polycondensation of all-*cis*-2,4,6,8-tetrahydroxy-2,4,6,8-tetraphenylcyclotetrasiloxane [25]. Note, the above fact supported the assumption that compound **1** was all-*cis-*[(Tol(HO)SiO]_4_.

The ^29^Si NMR spectra of polymers **6**–**8** in the δ region of −76.0~−82.0 ppm were characterized by a single signal at δ −79.9 ppm with ∆δ_1/2_ = 3.0 ppm. The signals at δ −67.0~−71.0 ppm specific for ClC_6_H_4_Si(OH)O units were present but were very small in the spectra of polymers **8** and **9** (Figure 7b).

**Scheme 1:**
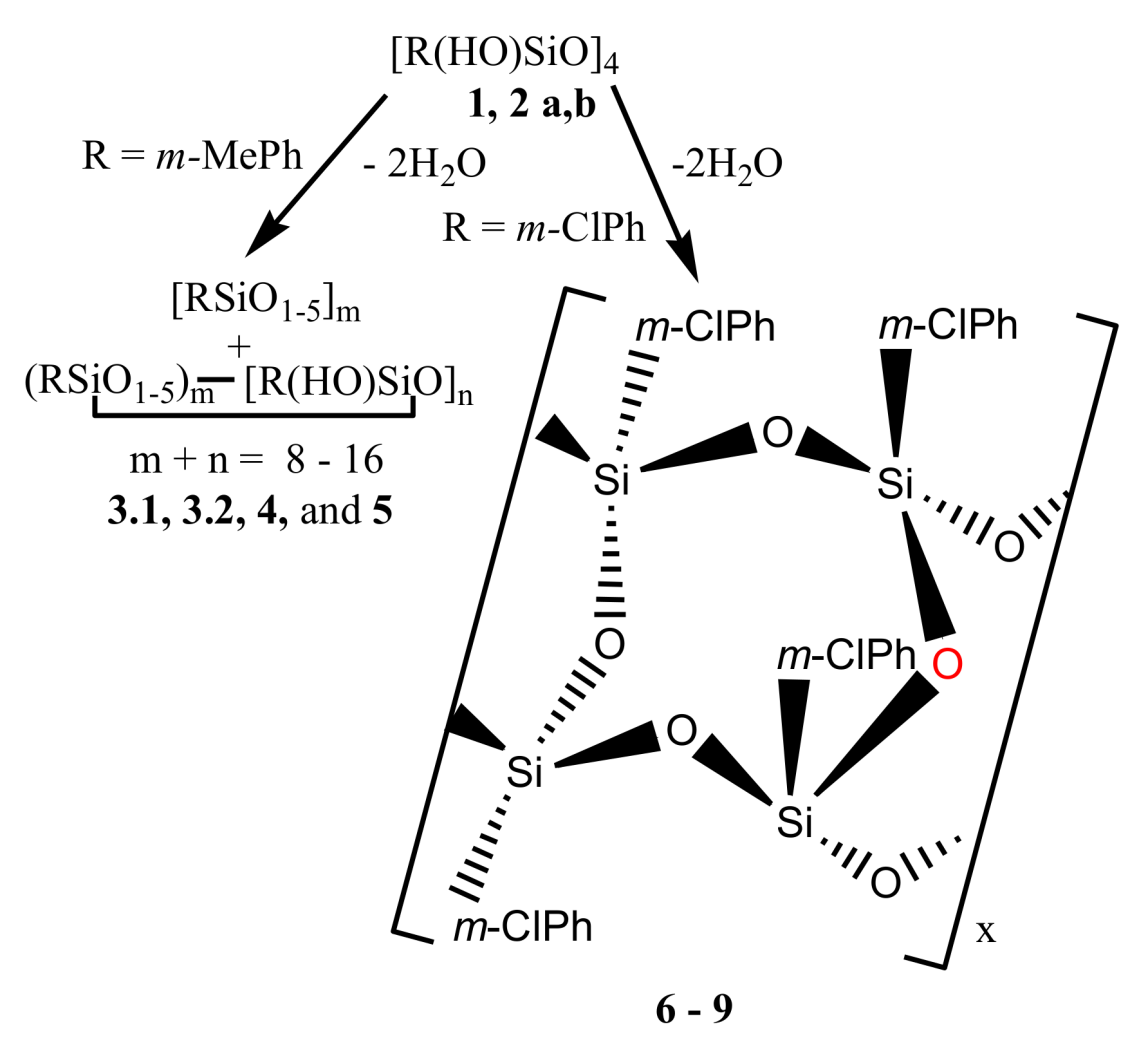
Thermal polycondensation of compounds **1** and **2a**,**b**.

Based on the data of ^29^Si NMR, IR, and mass spectra, polycondensation of compounds **1** and **2a**, **b** took place according to Scheme 1 with the formation of products **3.1**, **3.2**, **4**, and **5** and polymers **6**–**9**.

**Scheme 2:**
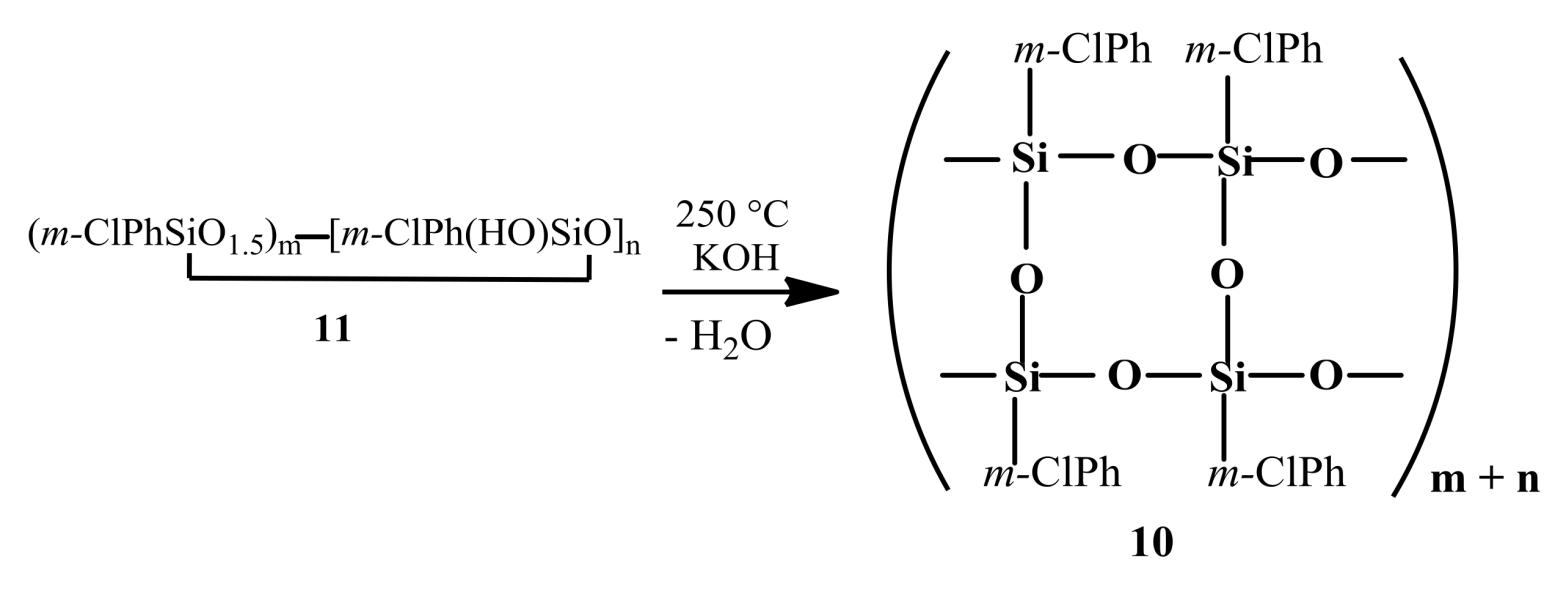
Anionic polymerization of prepolymer **11**.

A comparison was made of the polymer **8** unit configuration order with that of CL ladder poly-*m*-chlorophenylsilsesquioxanes (LPClPSSO) (**10**). This polymer was synthesized by anionic polymerization in the presence of KOH at 250 °C of a prepolymer (**11**) obtained by hydrolysis of *m*-chlorophenyltrichlorosilane (Scheme 2) [1].

After reprecipitation, polymer **10** had *M*_w_ = 5.66 × 10^5^, *M*_n_ = 1.15 × 10^5^, and *M*_w_/*M*_n_ = 4.90; [η] = 1.40 dL·g^−1^ (20 °C, benzene). The IR spectrum of polymer **10** was characterized by two bands at 1050~1055 and 1140~1145 cm^−1^ with comparable intensities (Figure 3, curve (*4*)). This confirmed a CL structure of the main siloxane chain in **10**. It is noteworthy that no OH bands were present in the spectrum. As mentioned above, polymer **8** showed a broadened singlet at δ –79.9 ppm in its ^29^Si NMR spectrum. At the same time, a multiplet with the main maxima at δ −79.3 and −81.5~−82.0 ppm was observed for polymer **10** (Figure 7b). With that, the ∆δ_1/2_ values for both polymers were the same. This splitting in the case of **10** was apparently due to different unit configurations in the polymer chain. These ^29^Si NMR data are in good accordance with those previously published for LPPSSO copolymers obtained by copolycondensation of all-*cis*- and *cis*-*trans*-*cis*-(tetrahydroxy)(tetraphenyl)cyclotetrasiloxanes in different proportions. Their ^29^Si NMR spectra showed two signals with δ −78.24 and −79.06 ppm [25].

Thus, sequences of units having different configurations were present in the structures of LPClPSSO synthesized by anionic polymerization, while the configuration of **2a** was retained throughout product **8** since only one singlet was detected in the ^29^Si NMR spectrum of this polymer.

Powder X-ray diffraction was used to determine the inter-chain distance (d_1_) in polymer **8**. It gave d_1_ = 14.40 Å. This value agrees with the data for low and high molecular-mass LPClPSSO obtained by anionic polymerization. However, this method is inappropriate for detecting configurational differences between chain units of CL LPClPSSO **8** and **10**.

## 4. Conclusions

Polycondensation of isomer **1** proceeds with the formation of oligomeric incomplete and complete cage-like compounds rather than CL polymers regardless of the reaction conditions and with and without MMT. Nevertheless, the polycondensation occurs more selectively in the presence of MMT than without it. As preliminarily shown, similar cage-like compounds were obtained by polycondensation of all-*cis* [Ph(HO)SiO]_4_. However, *cis*-isotactic CL LPPSSO of low molecular mass were also formed but only in the presence of MMT. In contrast to **1**, polycondensation of **2a** affords CL LPClPSSO in both the absence and presence of MMT. Nonetheless, the addition of MMT results in an increase in *M*_w_ of the polymer as compared with *M*_w_ of the polymer obtained in the absence of MMT.

Syndiotactic CL LPPSSO resulting from the polycondensation of *cis*-*trans*-*cis* [Ph(HO)SiO]_4_ in the presence of MMT that had similar spectral characteristics were previously reported. This suggests that such small substituents at phenyls as Me or Cl should not exert any significant effect, if at all, on the results of the polycondensation reactions conducted under identical conditions, provided the steric characteristics of monomers are the same. On the other hand, both the results of polymerization of tetraphenylcyclosiloxanes and those of polycondensation of [Ph(HO)SiO]_4_ where the corresponding monomers differed only in steric characteristics (see above) indicate that the steric characteristics of a monomer should substantially influence the direction of the reaction. This all means that the difference in the results for polycondensation of compounds **1** and **2a** most likely comes from the difference in their configuration.

Analysis of the ^29^Si NMR spectrum of LPClPSSO obtained by the polycondensation of compound **2a** (polymer **8**) and that of LPClPSSO prepared by anionic polymerization of prepolymer **11** in the presence of KOH (polymer **10**) showed the unit sequences in polymer **8** to be different from those of polymer **10** in configuration. With that, the configuration of 2a was retained throughout polymer **8**.

## Supplementary Materials

The following are available online at http://www.mdpi.com/2073-4360/10/4/422/s1. Notes 1–4, Additional mass spectra: Figure S1. PI APCI mass spectra of product **3.2** obtained after heating of a toluene solution of compound **1** in a quartz flask at 110 °C for: (**a**) 30 min; (**b**) 60 min; and (**c**) 90 min; Figure S2a,b. PI APCI mass spectra of product **3.1** obtained after heating of a toluene solution of compound **1** at atmospheric pressure in a molybdenum glass flask at 110 °C for 30 and 60 min, respectively; Figure S3. PI APCI mass spectra of product **5** obtained after heating of compound **1** at atmospheric pressure in the presence of MMT for 120 min (**a**) in anisole at 150 °C and (**b**) in toluene at 110 °C and after treatment of it with an excess of trimethylsilane; Figure S4. PI APCI mass spectra of product **4** obtained after heating of a ditolylmethane solution of compound **1** in vacuum at 95–97 °C for: (**a**) 30 min; (**b**) 60 min; (**c**) 90 min; and (**d**) 120 min; X-ray Structure Determination; Figure S5. Molecular structure of deca-*m*-tolylsilsesquioxane (**T_10_**) with thermal ellipsoids drawn at 30% probability level (the hydrogen atoms and disordered positions for the *m*-tolyl groups are omitted for clarity), Table S1. Selected bond lengths (Å) and angles (deg.) in **T_10_**.
