# Supplementary material for: A Study of the Polycondensation of (Tetrahydroxy)(Tetraaryl)Cyclotetrasiloxanes under Equilibrium and Non-Equilibrium Conditions in the Presence and Absence of Montmorillonite
Source: Polymers (Basel). 2018 Apr 9;10(4):422. doi: 10.3390/polym10040422 (PMC6415273; doi:10.3390/polym10040422)
Supplement: Supplementary file 1 [file polymers-10-00422-s001.pdf]

Supplementary Materials.

A study of polycondensaion of (tetrahydroxy)(tetraaryl)cyclotetrasiloxanes under equilibrium and non-equilibrium conditions in the presence and absence of montmorillonite

Natalia N. Makarova,* Yury I. Lyakhovetsky, Irina M.Petrova, Fedor M. Dolgushin, Nikolai S.Ikonnikov, Alexander S. Peregudov, Tatyana V. Strelkova and Zinaida S. Klemenkova

A.N. Nesmeyanov Institute of Organoelement Compounds of the Russian Academy of Sciences, 119991 GSP- 1 Moscow V-334, Russian Federation; yulyakh@ineos.ac.ru (Y.L.); impetr2013@yandex.ru (I.M.); fedya_xrlab (F.D.); ikonns@ ineos.ac.ru (N.I.); asp@ineos.ac.ru (A.P.); o.strelkova@gmail.com (T.S.); zklem@ineos.ac.ru (Z.K.)

*Correspondence: nmakar@ineos.ac.ru; Tel.: +7-499-1359243

(Polymers)

1. Notes

Note 1. Both molybdenum glass and MMT contain ions of metals, whereas quartz does not. Hence, if polycondensation in the presence of MMT had been conducted in quartz flasks, the effect of addition of MMT upon it could possibly have been attributed to catalysis by metal ions. To avoid this or at least make such suggestion less probable, only molybdenum glass flasks were used for the reactions carried out with the addition of MMT.

Note 2. In those cases where the peak abundances of the nominal mass ions were pronouncedly less than those simulated, contributions from the corresponding protonated molecules to the peak groups were probably present.

Note 3. The closed-cage compound deca-*m*-tolylsilsesquioxane ($\mathbf{T_{10}}$) discussed was synthesized by another method [S1] and its structure established by several analytical methods including X-ray analysis that showed no water molecule present (see below for its crystallographic data). At the same time, the PI APCI mass spectrum of $\mathbf{T_{10}}$ displayed the molecular ion with a water molecule added in the mass spectrometer. However, as emphasized in the main text, one cannot tell whether it was a hydrate or the hydrolysis occurred to give an ion with two hydroxyl groups.

Note 4. It occurred probably due to the fact that the molecular masses of products **6** - **8** were higher than 2000 Da, while the mass range of the mass spectrometer was bounded by this value.

2. Additional mass spectra.

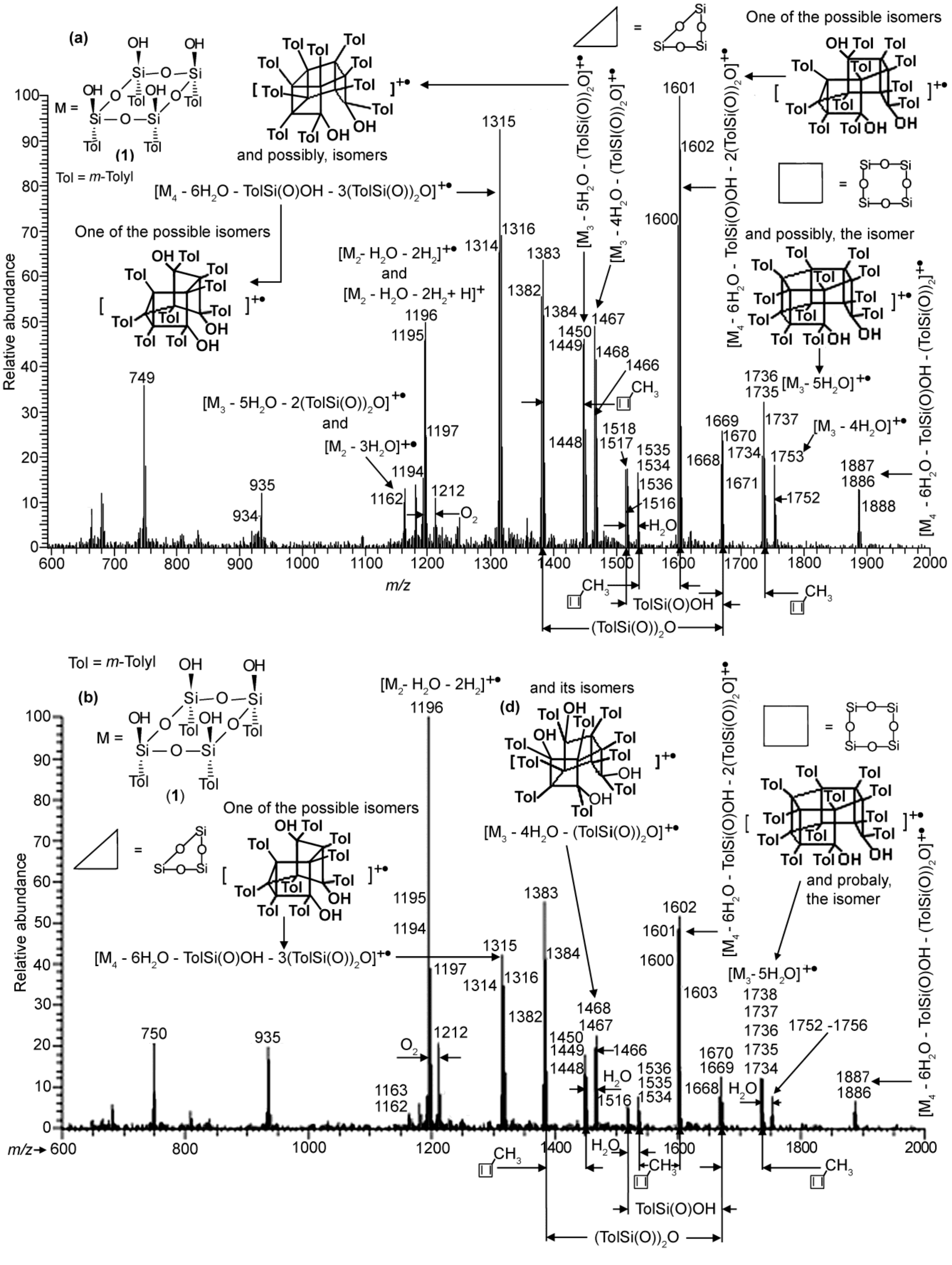
(a)
M =
(1)
Tol = m-Tolyl
and possibly, isomers
One of the possible isomers
[M4 - 6H2O - TolSi(O)OH - 3(TolSi(O))2O]+•
[M2- H2O - 2H2]+•
and
[M2 - H2O - 2H2+ H]+
[M3 - 5H2O - 2(TolSi(O))2O]+•
and
[M2 - 3H2O]+•
[M3 - 5H2O - (TolSi(O))2O]+•
[M3 - 4H2O - (TolSi(O))2O]+•
[M4 - 6H2O - TolSi(O)OH - 2(TolSi(O))2O]+•
[M4 - 6H2O - TolSi(O)OH - (TolSi(O))2]+•
and possibly, the isomer
[M3- 5H2O]+•
[M3 - 4H2O]+•
Relative abundance
m/z
TolSi(O)OH
(TolSi(O))2O
(b)
(d)
and its isomers
[M3 - 4H2O - (TolSi(O))2O]+•
and probaly, the isomer
[M3- 5H2O]+•

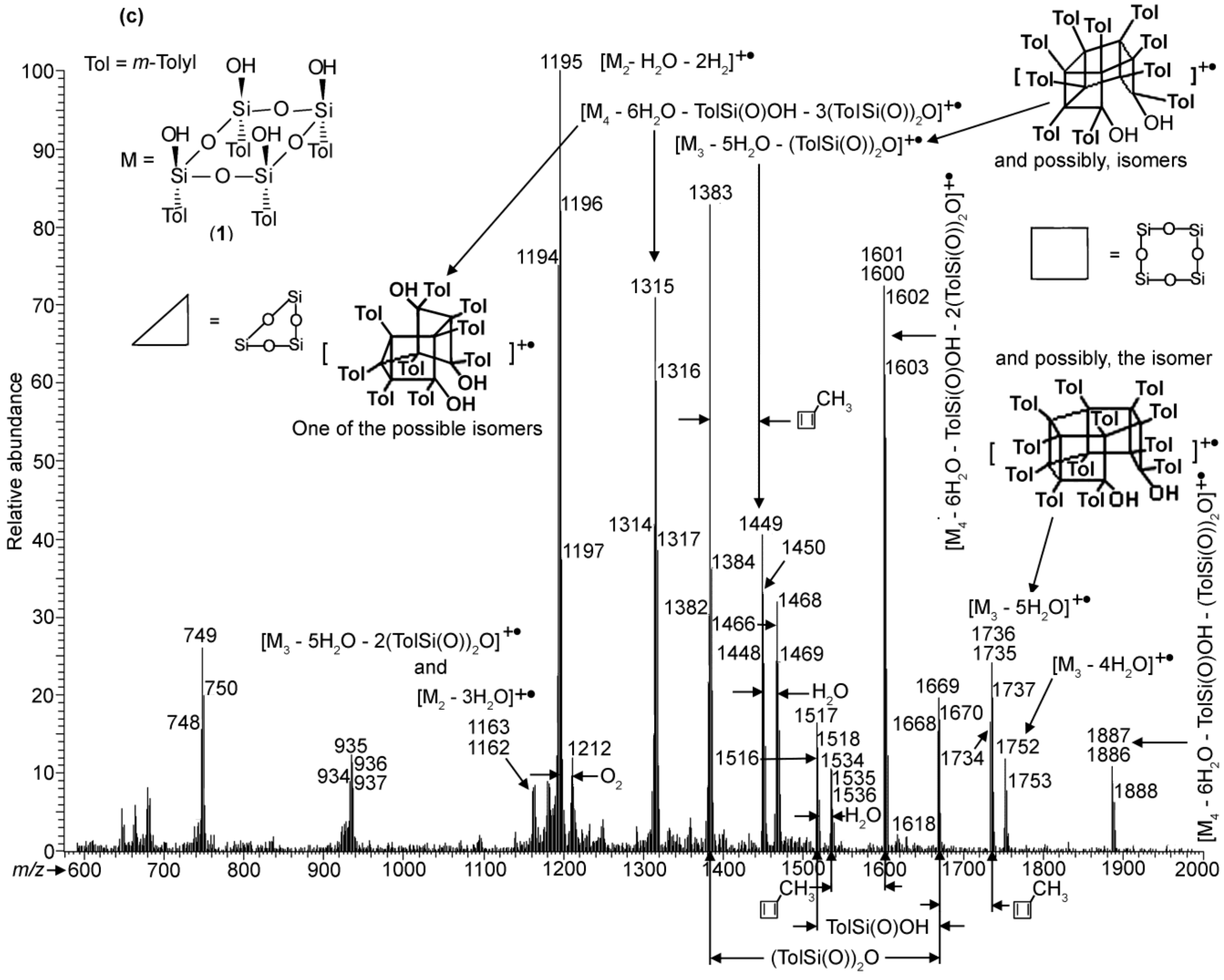

Figure S1. PI APCI mass spectra recorded of product **3.2** obtained after heating of a toluene solution of compound **1** in a quartz flask at 110 °C for: **(a)** 30 min, **(b)** 60 min, and **(c)** 90 min.

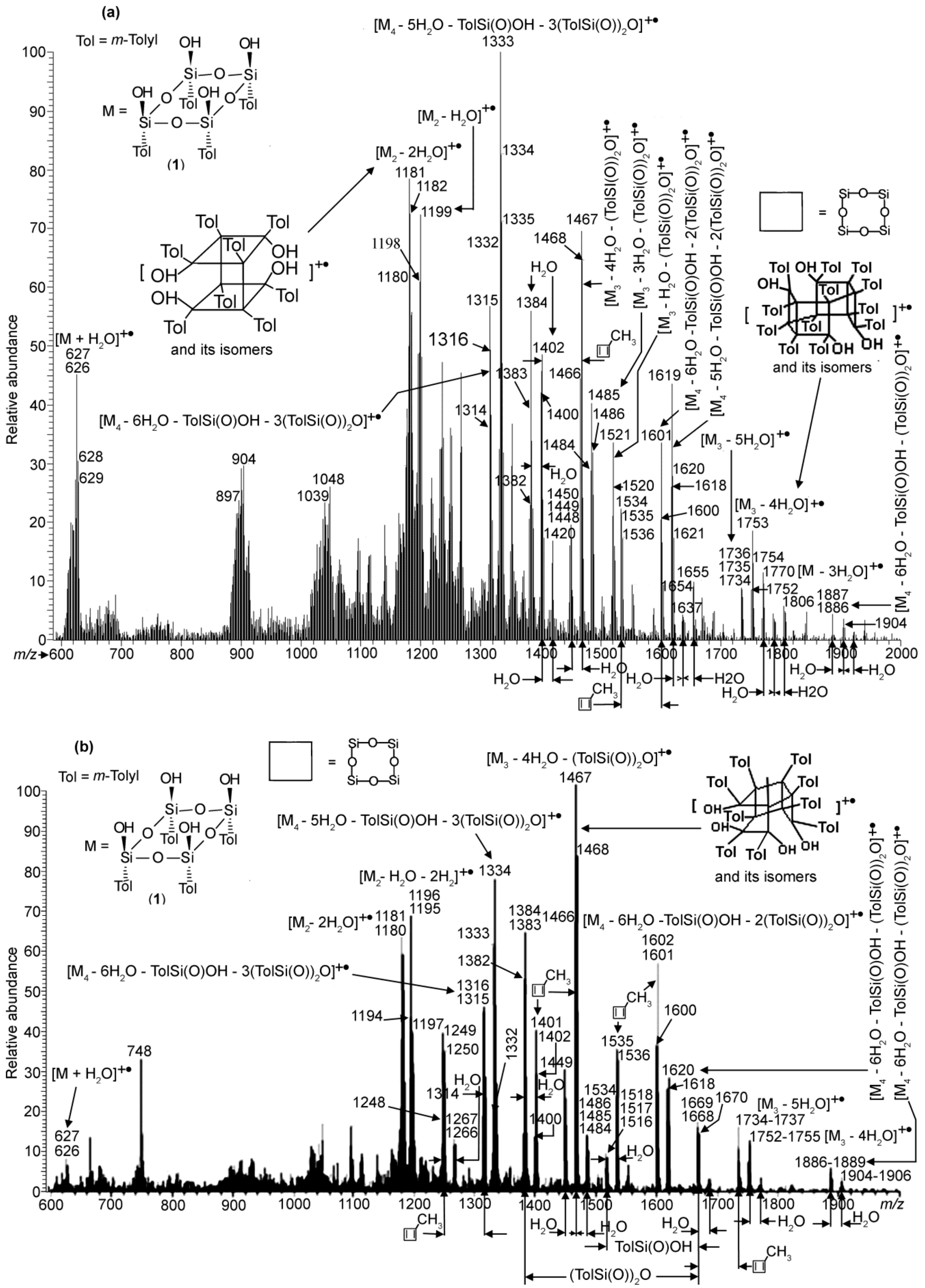

(a)
Tol = m-Tolyl
M =
(1)
and its isomers
Relative abundance
m/z
[M + H2O]+•
[M2 - 2H2O]+•
[M2 - H2O]+•
[M4 - 5H2O - TolSi(O)OH - 3(TolSi(O))2O]+•
[M4 - 6H2O - TolSi(O)OH - 3(TolSi(O))2O]+•
[M3 - 4H2O - (TolSi(O))2O]+•
[M3 - 3H2O - (TolSi(O))2O]+•
[M3 - H2O - (TolSi(O))2O]+•
[M4 - 6H2O - TolSi(O)OH - 2(TolSi(O))2O]+•
[M4 - 5H2O - TolSi(O)OH - 2(TolSi(O))2O]+•
[M3 - 5H2O]+•
[M3 - 4H2O]+•
[M - 3H2O]+•
[M4 - 6H2O - TolSi(O)OH - (TolSi(O))2O]+•
(b)
Tol = m-Tolyl
M =
(1)
and its isomers
Relative abundance
m/z
[M + H2O]+•
[M2 - 2H2O]+•
[M2 - H2O - 2H2]+•
[M4 - 5H2O - TolSi(O)OH - 3(TolSi(O))2O]+•
[M4 - 6H2O - TolSi(O)OH - 3(TolSi(O))2O]+•
[M3 - 4H2O - (TolSi(O))2O]+•
[M4 - 6H2O -TolSi(O)OH - 2(TolSi(O))2O]+•
[M3 - 5H2O]+•
[M3 - 4H2O]+•
[M4 - 6H2O - TolSi(O)OH - (TolSi(O))2O]+•
TolSi(O)OH
(TolSi(O))2O

Figure S2(a) and **(b)**: PI APCI mass spectra of product **3.1** obtained after heating of a toluene solution of compound **1** at atmospheric pressure in a molybdenum glass flask at 110 °C for 30 and 60 min, respectively.

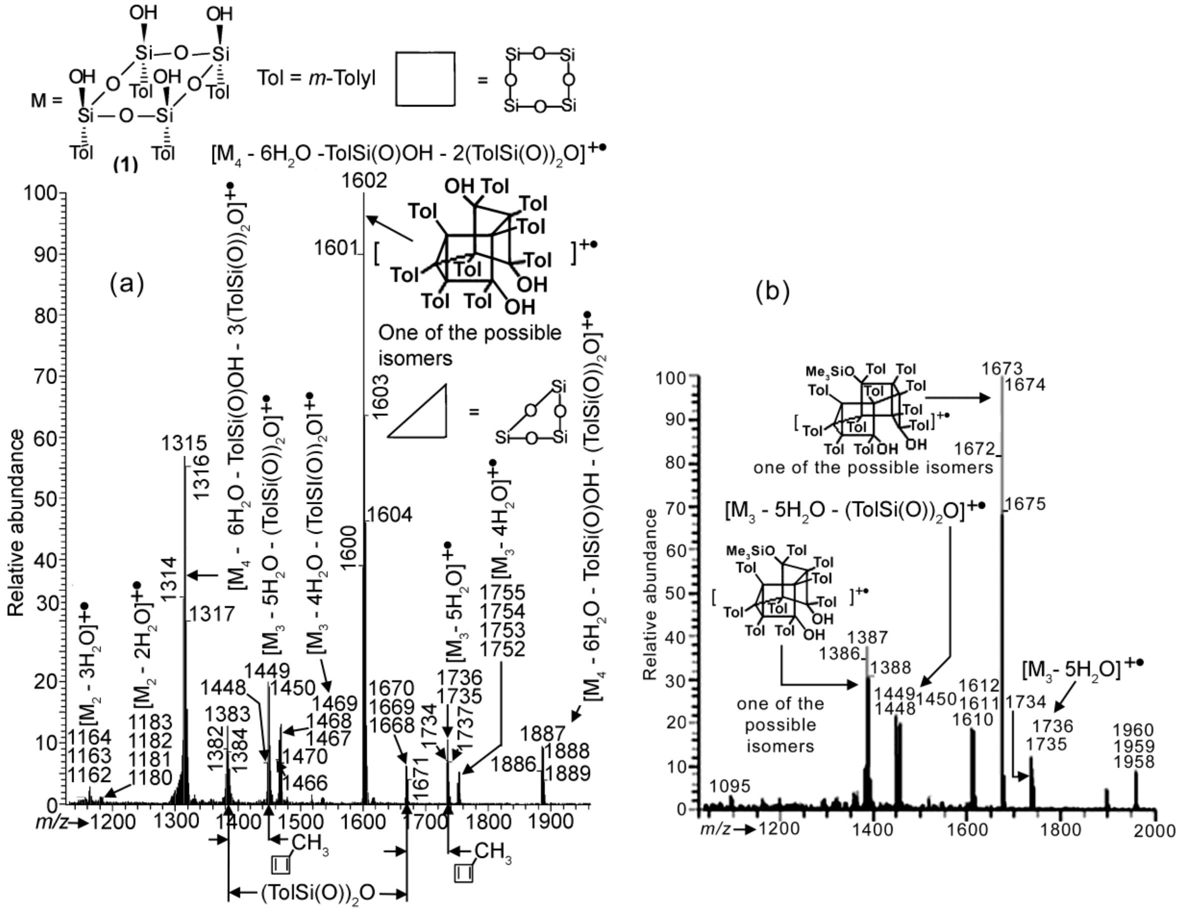

Figure S3. PI APCI mass spectra of product **5** formed after heating of compound **1** at atmospheric pressure in the presence of MMT for 120 min (**a**) in anisole at 150 °C and (**b**) in toluene at 110 °C and after treatment of it with an excess of trimethylsilane.

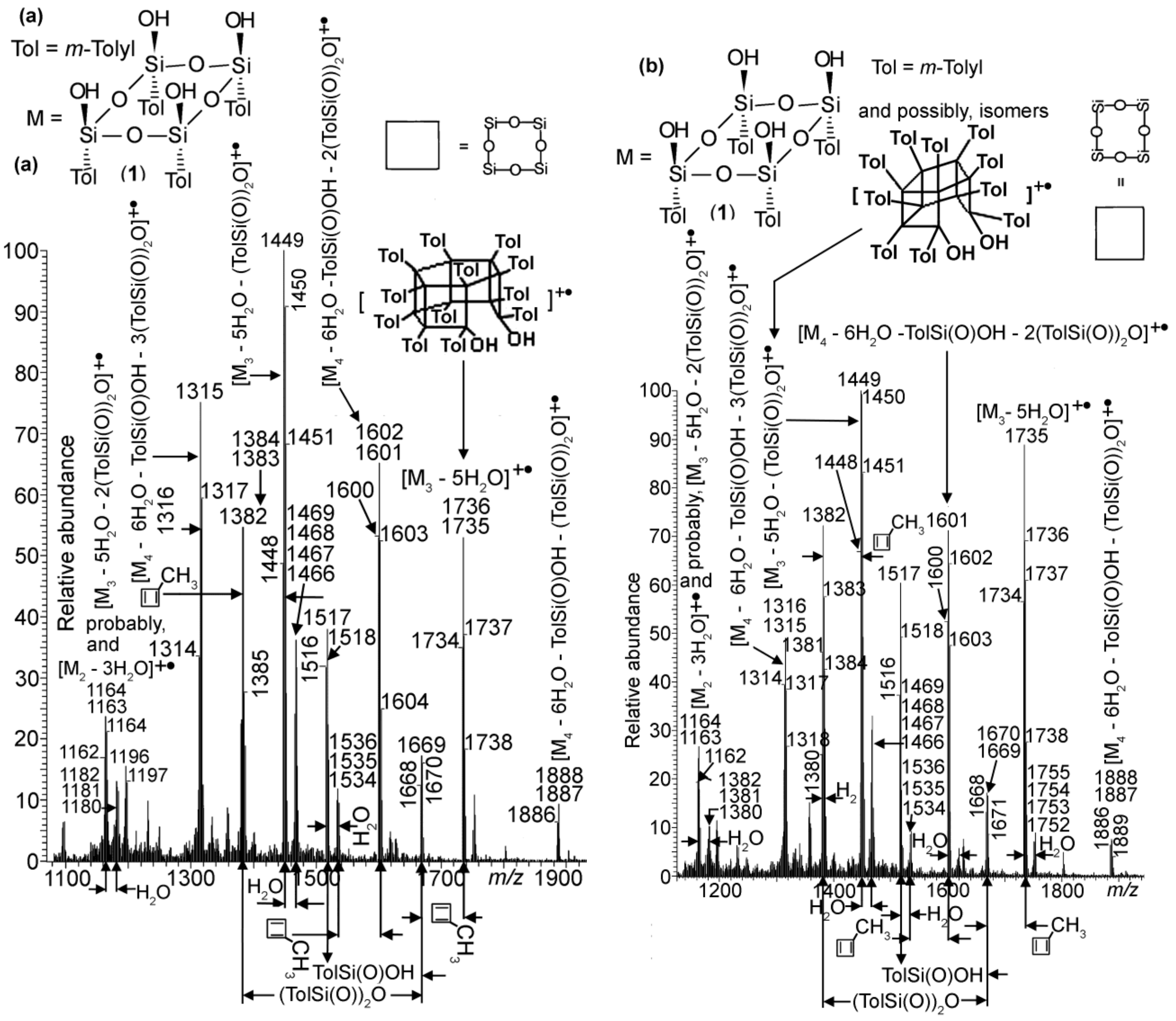

(a)
Tol = m-Tolyl
M =
(1)
Relative abundance
m/z
[M3 - 5H2O]+•
[M3 - 5H2O - (TolSi(O))2O]+•
[M4 - 6H2O -TolSi(O)OH - 2(TolSi(O))2O]+•
[M4 - 6H2O - TolSi(O)OH - (TolSi(O))2O]+•
[M4 - 6H2O - TolSi(O)OH - 3(TolSi(O))2O]+•
[M3 - 5H2O - 2(TolSi(O))2O]+•
probably, and
[M2 - 3H2O]+•
TolSi(O)OH
(TolSi(O))2O
(b)
Tol = m-Tolyl
and possibly, isomers

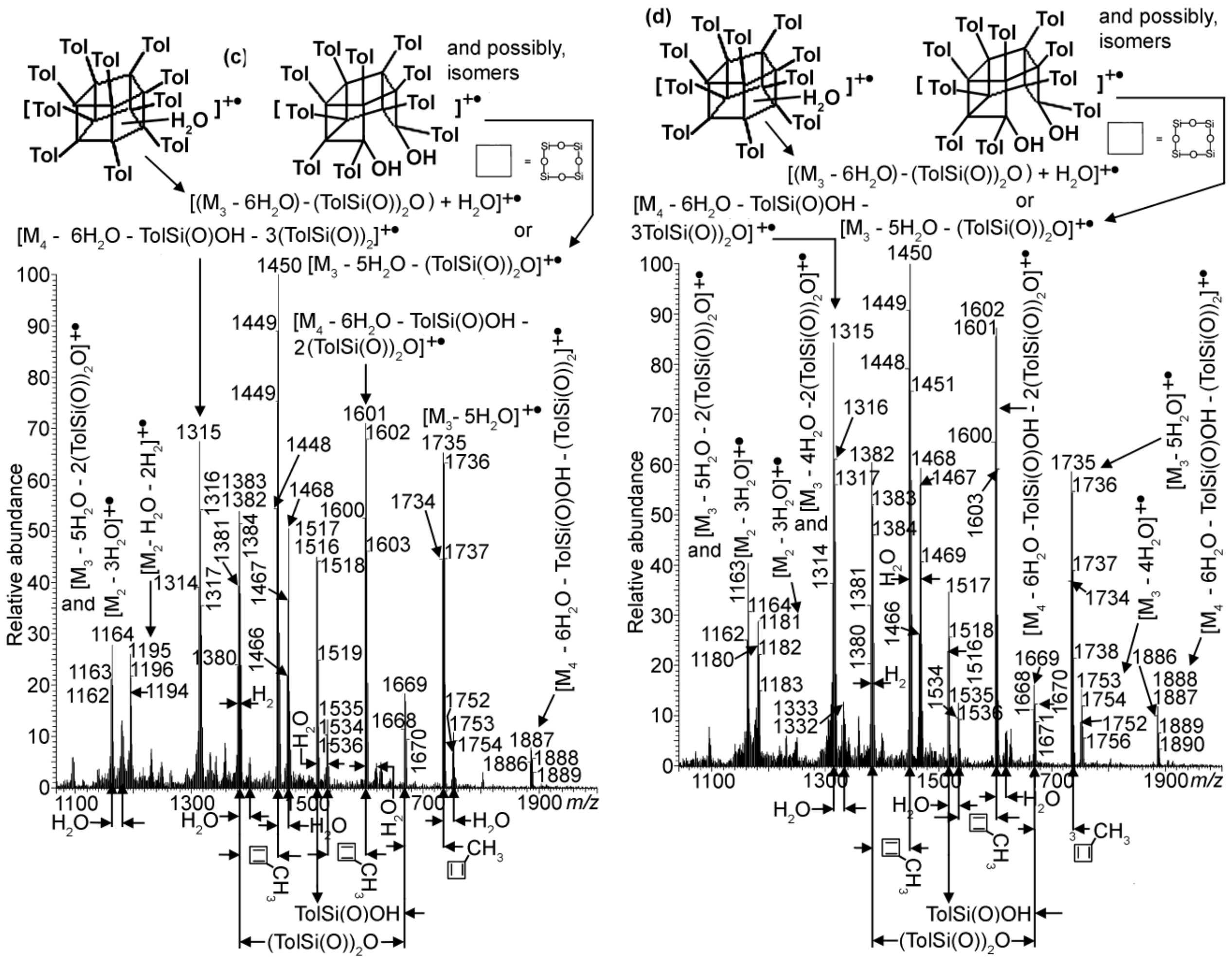

Figure S4. PI APCI mass spectra of product **4** obtained after heating of a ditolylmethane solution of compound **1** in vacuum at 95 – 97 °C for: **(a)** 30 min, **(b)** 60 min, **(c)** 90 min, and (**d**) 120 min.

3. X-ray Structure Determination.

Deca–*m*-tolylsilsesquioxane crystals ($C_{70}H_{70}O_{15}Si_{10}$, M = 1432.16, $\mathbf{T_{10}}$) are monoclinic, space group *C*2/c, at 120(2) K a = 28.3855(12), b = 14.0077(6), c = 22.1906(9) Å, β = 125.2180(8)°, V = 7208.3(5) Å^3, Z = 4, $d_{calc.}$ = 1.320 g/cm^3, μ = 2.46 cm^{-1}. Data were collected with a Bruker SMART APEX II diffractometer (λ(MoKα) = 0.71073 Å, ω-scan technique, T = 120(2) K, and a total of 7877 independent reflections (R_{int} = 0.0370) with $2\theta_{max}$ = 54.0° were collected and used in refinement). The APEX II software [S2] was used for collecting frames of data, indexing reflections, determination of lattice constants, integration of intensities of reflections, scaling and absorption correction, and the SHELXTL software [S3] was used for space group and structure determination, refinements, graphics, and structure reporting. The structure was solved by direct methods and refined by the full matrix least-squares technique against F^2 with the anisotropic thermal parameters for all non-hydrogen atoms. Four of the five independent *m*-tolyl substituents are highly disordered

and refined with the AFIX 66 restraint instruction. All hydrogen atoms were placed geometrically and included in the structure factors calculation in the riding motion approximation. The refinement converged to wR_2 = 0.1651 and GOF = 1.055 for all independent reflections (R_1 = 0.0540 was calculated against F for 5511 observed reflections with I > 2σ(I)).

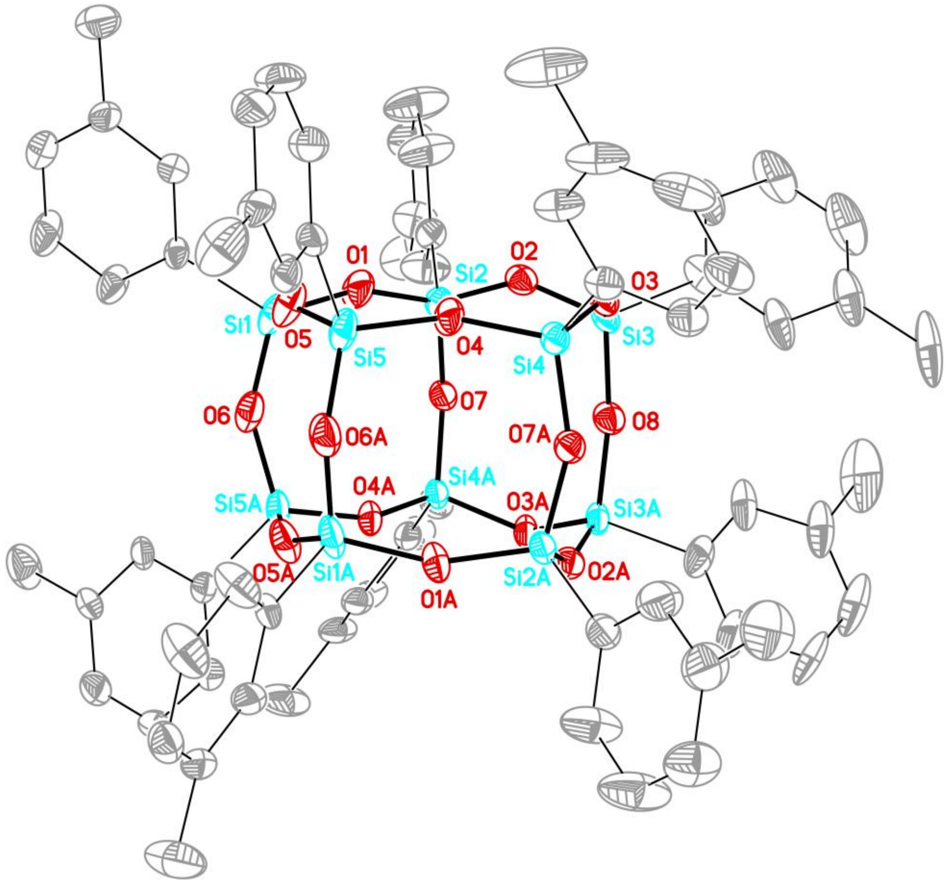

Figure S5. Molecular structure of deca-*m*-tolylsilsesquioxane (**T_{10}**) with thermal ellipsoids drawn at the 30% probability level (the hydrogen atoms and disordered positions for the *m*-tolyl groups are omitted for clarity).

Table S1. Selected bond lengths (Å) and angles (deg.) in **T_{10}**

Si(1)-O(1)	1.607(2)	O(1)-Si(1)-O(6)	108.73(12)
Si(1)-O(6)	1.618(3)	O(1)-Si(1)-O(5)	108.25(14)
Si(1)-O(5)	1.618(2)	O(6)-Si(1)-O(5)	110.57(13)
Si(2)-O(1)	1.614(2)	O(1)-Si(2)-O(2)	107.81(12)
Si(2)-O(2)	1.615(2)	O(1)-Si(2)-O(7)	110.25(11)
Si(2)-O(7)	1.616(2)	O(2)-Si(2)-O(7)	110.04(11)
Si(3)-O(8)	1.6091(8)	O(8)-Si(3)-O(2)	109.76(10)

Si(3)-O(2)	1.619(2)	O(8)-Si(3)-O(3)	107.12(9)
Si(3)-O(3)	1.6256(19)	O(2)-Si(3)-O(3)	110.05(10)
Si(4)-O(4)	1.612(2)	O(4)-Si(4)-O(7)#1	109.46(11)
Si(4)-O(7)#1	1.619(2)	O(4)-Si(4)-O(3)	107.70(11)
Si(4)-O(3)	1.6227(19)	O(7)#1-Si(4)-O(3)	109.70(10)
Si(5)-O(6)#1	1.609(2)	O(6)#1-Si(5)-O(5)	109.59(14)
Si(5)-O(5)	1.614(2)	O(6)#1-Si(5)-O(4)	108.78(11)
Si(5)-O(4)	1.618(2)	O(5)-Si(5)-O(4)	107.59(12)
O(6)-Si(5)#1	1.609(2)	Si(1)-O(1)-Si(2)	157.91(17)
O(7)-Si(4)#1	1.619(2)	Si(2)-O(2)-Si(3)	145.94(13)
O(8)-Si(3)#1	1.6091(8)	Si(4)-O(3)-Si(3)	138.75(12)
		Si(4)-O(4)-Si(5)	152.73(15)
		Si(5)-O(5)-Si(1)	151.33(15)
		Si(5)#1-O(6)-Si(1)	145.29(15)
		Si(2)-O(7)-Si(4)#1	153.01(14)
		Si(3)-O(8)-Si(3)#1	170.8(2)

The structure was registered in the database of the Cambridge Crystallographic Data Centre (CCDC) (http://www.ccdc.cam.ac.uk.”) under the number 1581185.The data can be obtained free of charge from it.

4. References

S1. Vogt, L.H.; Brown Jr., J. F. Crystalline Methylsilsesquioxanes. *Inorg. Chem.* **1963**, *2*, 189-192. DOI: 10.1021/ic50005a048.

S2. *APEX II software package*, Bruker AXS Inc., 5465, East Cheryl Parkway, Madison, WI 5317, 2005.

S3. Sheldrick, G.M. Crystal structure refinement with SHELXL, *Acta Crystallogr. C Struct. Chem.* **2015**, *71(Pt 1)***,** 3-8. DOI: 10.1107/S2053229614024218.
